# Identification and application of a pair of noncompeting monoclonal antibodies broadly binding to the nucleocapsid proteins of SARS-CoV-2 variants including Omicron

**DOI:** 10.1186/s12985-022-01827-w

**Published:** 2022-05-28

**Authors:** Bing Zhou, Lin Cheng, Shuo Song, Huimin Guo, Senlin Shen, Haiyan Wang, Xiangyang Ge, Lei Liu, Bin Ju, Zheng Zhang

**Affiliations:** 1 Institute for Hepatology, National Clinical Research Center for Infectious Disease, Shenzhen Third People’s Hospital, The Second Affiliated Hospital, School of Medicine, Southern University of Science and Technology, Shenzhen, 518112 Guangdong Province China; 2 Guangdong Key Laboratory for Anti-Infection Drug Quality Evaluation, Shenzhen, 518112 Guangdong Province China; 3 Shenzhen Research Center for Communicable Disease Diagnosis and Treatment of Chinese Academy of Medical Science, Shenzhen, 518112 Guangdong Province China

**Keywords:** SARS-CoV-2 variants, Delta, Omicron, Nucleocapsid protein, Monoclonal antibody

## Abstract

The SARS-CoV-2 nucleocapsid protein (NP) is an important indicator for the virus infection, highlighting the crucial role of NP-specific monoclonal antibodies (mAbs) used in multiple biochemical assays and clinical diagnosis for detecting the NP antigen. Here, we reported a pair of noncompeting human NP-specific mAbs, named P301-F7 and P301-H5, targeting two distinct linear epitopes on SARS-CoV-2 or SARS-CoV. We evaluated the application of P301-F7 in the analysis of enzyme linked immunosorbent assay, western blot, flow cytometry, immunofluorescence, and focus reduction neutralization test. We for the first time report a broad mAb effectively recognizing various live viruses of SARS-CoV-2 variants including Alpha, Beta, Delta, and Omicron, indicating a wide range of application prospects.

**Supplementary Information:**

The online version contains supplementary material available at 10.1186/s12985-022-01827-w.

## Introduction

The coronavirus disease 2019 (COVID-19) caused by severe acute respiratory syndrome coronavirus 2 (SARS-CoV-2) was first reported in late 2019 and early 2020 [1]. It was the third coronavirus disease in the twenty-first century after SARS-CoV in 2002 [2] and Middle East respiratory syndrome coronavirus (MERS-CoV) in 2012 [3]. So far, there has been over 480 million confirmed cases, resulting in more than 6 million deaths worldwide. Since the beginning of 2021, various SARS-CoV-2 variants have been emerging one after another, such as Alpha, Beta, Gamma, Delta, and especially current Omicron, which carried a lot of mutations compared with the Wuhan reference strain (wild-type, WT).

SARS-CoV-2 is a member of the sarbecovirus subgenus. It contains 14 main open reading frames and encodes 4 structural proteins including surface spike protein, envelope protein, integral membrane protein, and nucleocapsid protein (NP) [4]. Among them, spike protein mediates the interaction of virus with cellular receptor (angiotensin-converting enzyme 2, ACE2) and triggers subsequent cell membrane fusion for the viral entry [5]. Several studies have shown that mutations located in spike protein contributed to the escape from the neutralizing antibodies (nAbs), making the SARS-CoV-2 variants a problem to be concerned worldwide [6–9]. All along, researchers have also been paying close attention to the mutations in the NP [10, 11]. The SARS-CoV-2 NP is a highly immunogenic and abundantly expressed protein during the viral infection, making it as a suitable target for antigen detection [12, 13]. As we all know, the NP-specific antibodies play a crucial role in developing test reagents. Currently, several monoclonal antibodies (mAbs) have been identified from both immunized mice and rabbits and convalescent COVID-19 patients, and some of the mAbs are also able to recognize the mutated NP of SARS-CoV-2 variants [14–17]. However, compared with a large number of spike-specific mAbs, the quantity of anti-NP mAbs is generally limited, whose functions and features have not been clearly characterized. More importantly, it is still unknown that whether these applied mAbs could bind to the live virus of variants, especially for the detection of SARS-CoV-2 Delta and Omicron variants dominating the current wave of the COVID-19 pandemic around the world.

In this study, we isolated a pair of noncompeting NP-specific mAbs from a convalescent COVID-19 individual. P301-F7 and P301-H5 bound well to the WT and mutated NPs, as well as SARS-CoV NP, which recognized two distinct linear epitopes. We also validated P301-F7 in the analyses of enzyme linked immunosorbent assay (ELISA), western blot (WB), flow cytometry, immunofluorescence, and focus reduction neutralization test (FRNT). Most of all, to our knowledge, we first provided a NP-specific mAb for the detection of SARS-CoV-2 variant live viruses including Alpha, Beta, Delta, and Omicron variants, which could be suitable for various applications in the future.

## Materials and methods

### Blood samples

The donor P301 was infected with WT SARS-CoV-2. She had been running a fever for 6 days and then admitted to Shenzhen Third People’s Hospital at January 31, 2020. She was discharged from the hospital at February 14, 2020. Blood samples were collected at February 12, 2020 in the convalescent period. Plasma samples were stored at − 80 °C and PBMCs were maintained in freezing medium and stored in liquid nitrogen in the Biobank of Shenzhen Third People’s Hospital.

### Flow cytometry

PBMCs from the COVID-19 patient were collected and incubated with the LIVE/DEAD™ Fixable Dead Cell Stain reagent (Thermo Scientific) to exclude dead cells. Cells were then stained with His-tagged WT SARS-CoV-2 NP (Sino Biological) and the fluorescent-labeled antibodies including CD19-PE/Cy7, CD3-Pacific Blue, CD8-Pacific Blue, CD14-Pacific Blue, CD27-APC/Cy7, IgG-FITC (BD Biosciences), followed by incubated with APC- and PE- labeled His-specific antibodies (Abcam). NP-specific single B cells were gated as CD19 + CD3-CD8-CD14-CD27 + IgG + NP + and sorted into 96-well PCR plates containing lysis buffer (Invitrogen) using a FACSAriaII Flow Cytometer (BD Biosciences). Plates were then snap-frozen on dry ice and stored at − 80 °C until RT reaction.

For intracellular staining, 293 T cells transfected by NP expression vectors were fixed and permeabilized using Fixation/Permeabilization Solution Kit (BD Biosciences), stained with NP-specific mAbs (P301-F7 or P301-H5) or the polyclonal antibody (pAb) (Sino Biological), followed by staining with APC-conjugated secondary antibody (Life Technologies). After washing, cells were resuspended and subjected to acquisition with a FACSCalibur Flow Cytometer (BD Biosciences). Data were analyzed with FlowJo software V10.6 (BD Biosciences).

### Single B cell PCR, cloning, and expression of mAbs

To amplify IgG variable genes, we first carried out the RT reaction in the 96-well PCR plate. Then heavy chain and light chain genes were separately amplified, sequenced, synthesized, and cloned into the backbone of antibody expression vectors containing the constant regions of human IgG1 (Sangon Biotech). MAbs were produced by transient transfection of 293 F cells (Life Technologies) with equal amounts of paired heavy and light chain plasmids, and purified by affinity chromatography using Protein A beads columns (Senhui Microsphere Tech) according to the manufacturer’s protocol.

### Enzyme-linked immunosorbent assay (ELISA)

The native recombinant nucleocapsid proteins (NPs) of SARS-CoV, SARS-CoV-2 or variants, MERS-CoV, HKU1, OC43, NL63, and 229E (Sino Biological) (2 μg/mL) or denatured by treatment with the denaturing buffer containing 0.5% SDS and 40 mM DTT [18] (New England Biolabs) at 95 °C for 10 min were coated into 96-well half-area plates overnight at 4 °C. The plates were then blocked with blocking buffer (PBS containing 4% skim milk) at 37 °C for 1 h. Five-fold or three-fold serial-diluted NP-specific mAbs or Rabbit pAb against NP of SARS-CoV-2 (Sino Biological) were added to the plates in duplicate and incubated for 1 h at 37 °C. And then HRP-conjugated Goat anti-Human IgG (ZSGB-BIO) or Goat anti-Rabbit IgG (TransGen Biotech) secondary antibody was added to the plates and incubated at 37 °C for 1 h. For competitive ELISA, after blocking, serially diluted P301-F7, P301-H5, and human IgG1 were mixed with HRP-conjugated P301-F7, and then added to the plates and incubated at 37 °C for 1 h. The enzymatic reaction was developed with TMB substrate (BD Biosciences) and stopped by 2 M H_2_SO_4_. The optical density was measured at 450 nm (OD. 450 nm) with a Varioskan™ LUX Multimode Microplate Reader (Thermo Scientific).

### Binding analysis by surface plasmon resonance (SPR)

The binding assays of mAbs to NPs of SARS-CoV-2 and SARS-CoV were performed using the Biacore 8 K system (GE Healthcare). Specifically, one flow cell of the CM5 sensor chips were covalently coated with the NPs (Sino Biological) in 10 mM sodium acetate buffer (pH 5.0) for a final RU (response units) around 250, whereas the other flow cell was left uncoated and blocked as a control. All the assays were run at a flow rate of 30 µL/min in HBS-EP buffer (10 mM HEPES pH 7.4, 150 mM NaCl, 3 mM EDTA and 0.05% Tween-20). Serially diluted antibodies were injected for 60 s respectively and the resulting data were fit in a 1:1 binding model with Biacore Evaluation software (GE Healthcare). Every measurement was performed three times and the individual values were used to produce the mean affinity constant and standard deviation.

### Structure model prediction

The complete SARS-CoV-2 NP contains 419 amino acids, but a complete structure has not been resolved as it contains many flexible domains. To predict the model of the full-length SARS-CoV-2 NP, Coot was used to connect existing crystal structures of N-terminal domain (NTD, aa44-174, PDB code: 7CDZ) and C-terminal domain (CTD, aa255-364, PDB code: 7CE0), and the remaining parts were labeled with loops. Then the model was served as a template to predict the complete structure of SARS-CoV-2 NP using AlphaFold2 (https://alphafold.ebi.ac.uk). The image was depicted using PyMOL visualization software (http://www.pymol.org).

### Western blot analysis

For recombinant protein, 2 μg His-tagged NPs of SARS-2-CoV, SARS-CoV, and MERS-CoV (Sino Biological) were loaded onto 10% sodium dodecyl sulphate polyacrylamide gel electrophoresis (SDS-PAGE), transferred to polyvinylidene difluoride (PVDF) membrane using the Mini-PROTEAN® Tetra System (Bio-Rad). After blocking with 5% skim milk for 1 h at room temperature, membranes were incubated with HRP-conjugated mouse anti-His mAb (Sangon Biotech) or P301-F7, P301-H5 overnight at 4 °C, followed by incubation with HRP-conjugated Goat anti-Human IgG (ZSGB-BIO) for 1 h at room temperature. The proteins were visualized with Chemiluminescent Substrate (Thermo) and a ChemiDoc™ MP Imaging System (Bio-Rad).

### Immunofluorescence

Vero E6 cells were infected with SARS-CoV-2 at a MOI of 0.01 in the treatment of different doses of Remdesivir. At 48 h post-infection, cells were fixed with 4% paraformaldehyde for 30 min at RT, permeabilized with Perm/Wash (BD Biosciences) containing 0.1% Triton X-100 for 10 min at RT. After washing, cells were stained with 2 μg/mL NP-specific mAb (P301-F7) or pAb (Sino Biological) for 1 h at RT, followed by staining with Alexa Fluor® 488-conjugated secondary antibody (Invitrogen) for 1 h at RT. After washing, cells were incubated with Hoechst 33258 (Life Technologies). Microscopic images were obtained under a digital inverted microscope EVOS (Life technologies).

### Focus reduction neutralization test

SARS-CoV-2 focus reduction neutralization test (FRNT) was performed in a certified Biosafety level 3 lab. Antibodies were three-fold serial diluted, mixed with equal volume of SARS-CoV-2 or variants in U-bottom 96-well plates and incubated for 60 min at 37 °C. The mixture (containing 200 focus forming unit of live virus) were then transferred to 96-well plate seeded with Vero E6 cells and allowed absorption for 1 h at 37 °C before removed. After washing, the overlay media (MEM containing 1.6% Carboxymethylcellulose, 2% fetal bovine serum) was added and then cells were incubated at 37 °C for 24 h. After removing the overlay media, cells were fixed with 4% paraformaldehyde solution, permeabilized with Perm/Wash buffer (BD Biosciences) containing 0.1% Triton X-100, incubated with HRP-conjugated P301-F7. The reactions were developed with KPL TrueBlue Peroxidase substrates (Seracare Life Sciences). The numbers of SARS-CoV-2 or variants foci were calculated using an EliSpot reader (Cellular Technology Ltd).

### SARS-CoV-2 WT and variant strains

The SARS-CoV-2 original strain (WT) and Alpha, Delta, Omicron variant strains were separated in our biosafety level 3 lab, and the Beta strain was kindly gifted from Guangdong Provincial Center for Disease Control and Prevention, Guangdong Center for Human Pathogen Culture Collection (GDPCC). The whole genome sequences of WT and Alpha, Delta variant strains have been deposited in the Genome Warehouse in National Genomics Data Center, Beijing Institute of Genomics, Chinese Academy of Sciences/China National Center for Bioinformation, whose accession numbers were showed below and had been publicly accessible at https://ngdc.cncb.ac.cn/gwh. The clade of the isolated Omicron variant was determined using the Nextclade web application (https://clades.nextstrain.org/) and the sequence information has not been uploaded yet.

WT strain:

Beta/Shenzhen/SZTH-003/2020, EPI_ISL_406594 at GISAID;

Alpha strain:

SZTH008, Accession No. GWHBFWX01000000;

Beta strain:

GDPCC-nCoV84, Accession No. GWHBDSE01000000;

Delta strain:

SZTH012, Accession No. GWHBFWZ01000000.

### Animo acid sequences of antibodies

P301-F7 heavy chain:

QLQLQESGPGLVKPSETLSLTCTVSGGSISSTSYYWGWIRQPPGKGLEWIGSIYYSGSTYYNPSLKSRVTISVDTSKNQFSLKLSSVTAADTAVYYCARFSLYCSSTSCYENWFDPWGQGTLVTVSS

P301-F7 light chain:

QSVLTQPPSVSAAPGQKVTISCSGSSSNIGNNYVSWYQQLPGTAPKLLIYDNNKRPSGIPDRFSGSKSGTSATLGITGLQTGDEADYYCGTWDSSLSAGQVVFGGGTKLTVL

P301-H5 heavy chain:

EVQLVESGGGLVQPGGSLKLSCAASGFTFSGSAMHWVRQASGKGLEWVGRIRSKANSYATAYAASVKGRFTISRDDSKNTAYLQMNSLKTEDTAVYYCNFRGAFDYWGQGTLVTVSS

P301-H5 light chain:

SYELTQPPSVSVSPGQTARITCSGDALPKQYAYWYQQKPGQAPVLVIYKDSERPSGIPERFSGSSSGTTVTLTISGVQAEDEADYYCQSADSSGTYVVFGGGTKLTVL

## Results

**Fig. 1:**
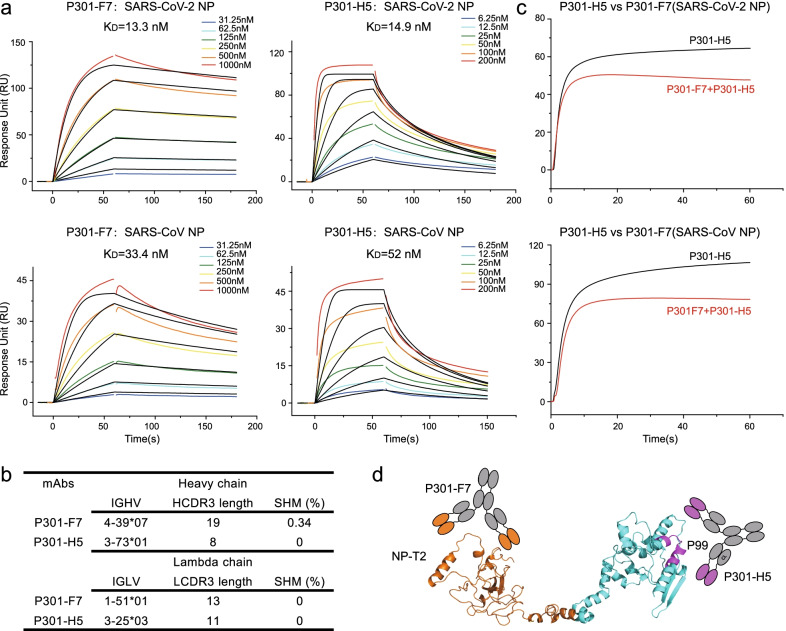
A pair of noncompeting mAbs binding to the SARS-CoV-2 and SARS-CoV NPs. **a** Binding affinities of P301-F7 and P301-H5 to NPs of SARS-CoV-2 (upper) and SARS-CoV (lower) by SPR. The data are means of three independent experiments. **b** Gene family analysis of P301-F7 and P301-H5. **c** P301-F7 and P301-H5 bound to two distinct epitopes on the SARS-CoV-2 NP (upper) and SARS-CoV NP (lower) by competition SPR. The experiment was performed twice and one representative result was shown. **d** Predicted model of the SARS-CoV-2 NP recognized by P301-F7 (brown) and P301-H5 (purple). The model was predicted using Alphafold 2.0 based on the N-terminal and C-terminal molecular structures (PDB codes: 7CDZ and 7CE0)

Our group had established a systemic Biobank of COVID-19 patients, so we performed the isolation of human-derived mAbs against SARS-CoV-2 NP. We first measured the binding activity of plasma from a convalescent individual, P301, which displayed a high titer of NP-specific IgG (Additional file 1: Figure S1a). Then we used the SARS-CoV-2 NP as a bait to sort specific single memory B cells by flow cytometry (Additional file 1: Figure S1b). Subsequently, single cell PCR, sequencing, and protein expression were performed to isolate mAbs as previous studies [5, 18]. Five mAbs were found to be able to bind to SARS-CoV-2 NP in different degrees, and P301-F7 and P301-H5 also recognized SARS-CoV NP well, yet none of which crossly targeted MERS-CoV NP (Additional file 1: Figure S1c). By the surface plasmon resonance (SPR), we measured the binding affinities of P301-F7 and P301-H5, whose dissociation constants (K_D_s) to SARS-CoV-2 NP and SARS-CoV NP were 13.3 nM, 14.9 nM, 33.4 nM, and 52.0 nM, respectively (Fig. 1a). The heavy chains of P301-F7 and P301-H5 were derived from IGHV4-39 and 3-73, and light chains belonged to IGLV1-51 and 3-25, respectively, with different loop lengths of complementarity determining region 3 (CDR3) and low degrees of somatic hypermutation (SHM) (Fig. 1b). Using the competition SPR and ELISA, we predicted their binding epitopes (Fig. 1c and Additional file 1: Figure S1d). P301-F7 and P301-H5 were a pair of noncompeting mAbs crossly targeting both SARS-CoV-2 and SARS-CoV NPs.

To further identify the epitopes recognized by P301-F7 and P301-H5, we first measured their binding activities to native and denatured SARS-CoV-2 NPs. As shown in Additional file 1: Figure S2a, though the denatured buffer strongly unfolded the conformation of NP, both mAbs could bind to it as well as what they did to native NP, indicating that P301-F7 and P301-H5 recognized the linear epitopes. To further narrow the scope of possible epitopes, we constructed full-length and two C-terminal truncated SARS-CoV-2 NPs and expressed them in 293 T cells by the transient transfection, respectively (Additional file 1: Figure S2b and Additional file 1: Figure S2c). P301-F7 recognized the N-terminal domain of NP (M1-M210) and P301-H5 bound to the C-terminal domain (E280-A419) by the flow cytometry analysis. Finally, by the screening of single peptide in ELISA, we identified that the P99 peptide (393-TLLPAADLDDFSKQL-407) was the key epitope recognized by P301-H5. However, none of tested single peptides (P1 to P51, covering M1 to G215 of NP) could strongly react with P301-F7 (Additional file 1: Figure S2d), which may be caused by the truncation of linear epitopes. Molecular modeling of the SARS-CoV-2 NP revealed that the binding epitope of P301-H5 was located on the surface of NP and spatially separated from the target region of P301-F7 in the structure model (Fig. 1d), suggesting the possibility to use this pair of noncompeting mAbs to develop assays for sandwich detection of antibodies. To investigate that, we performed the sandwich ELISA to detect SARS-CoV and SARS-CoV-2 NPs using P301-F7 and HRP conjugated P301-H5. The results showed that this pair of mAbs could effectively detect SARS-CoV and SARS-CoV-2 NPs in an antigen dose-dependent manner, respectively (Additional file 1: Figure S3).

**Fig. 2:**
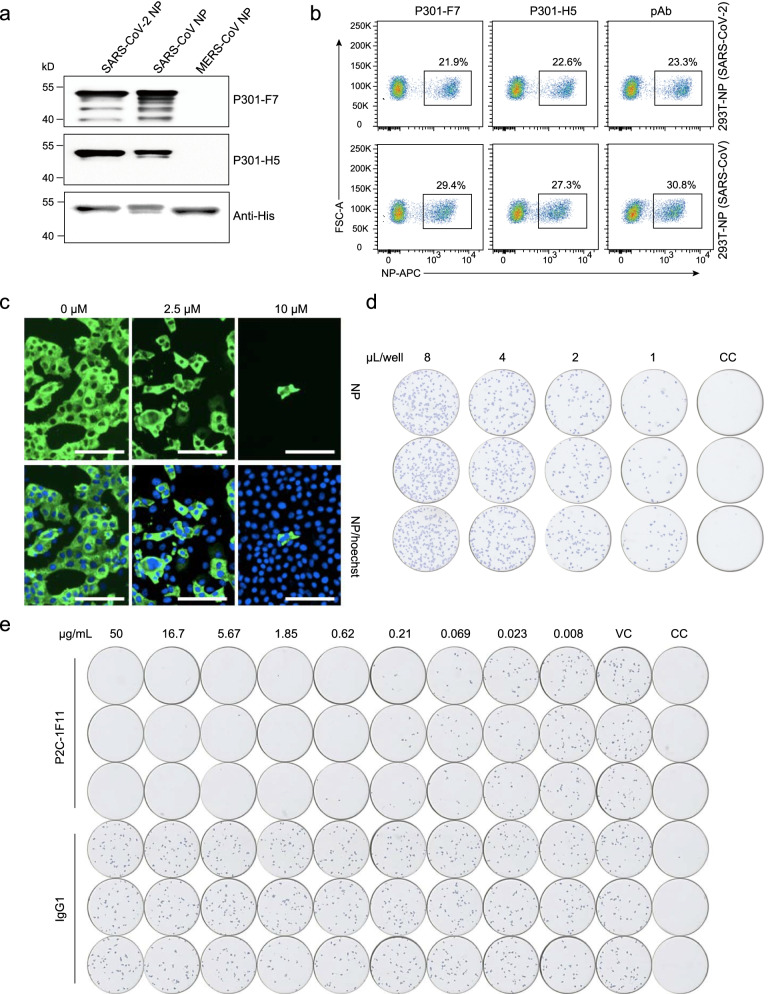
The application of P301-F7 and P301-H5 in the detection of NPs in multiple biochemical assays. **a** Western blot analysis of the binding activities of P301-F7 and P301-H5 to NPs of SARS-CoV-2, SARS-CoV, and MERS-CoV, respectively. The His-tag at C-terminus of each NP detected by the His-specific antibody is used here as a positive control in the assay. **b** Flow cytometry analysis of the binding activities of P301-F7 and P301-H5 to the SARS-CoV-2 and SARS-CoV NPs. Commercial polyclonal antibodies of one kind are used here as a positive control. **c** Immunofluorescence analysis of the inhibition effect of SARS-CoV-2 live virus by Remdesivir. Intracellular expression of NP was detected by staining Vero E6 cells infected with SARS-CoV-2 by P301-F7. The nuclei is stained with Hoechst. **d** The immune spot assay analysis of P301-F7 for recognizing the Vero E6 cells infected with SARS-CoV-2 live virus represented by blue spots. **e** The immune spot assay analysis of the inhibition efficiency of SARS-CoV-2 live virus in Vero E6 cells by P2C-1F11. IgG1 is a negative control of irrelevant antibody. VC: virus control without antibody. CC: cell control without virus and antibody. Each experiment was performed twice and one representative result was shown

To explore the more potential applications of P301-F7 and P301-H5, we performed immunoblot analysis with NPs of different coronaviruses. Both mAbs could detect the main 46-kDa protein band consistent with the molecular mass of SARS-CoV-2 and SARS-CoV NPs, yet did not crossly react with MERS-CoV NP (Fig. 2a). Similar to the commercial polyclonal antibodies, P301-F7 and P301-H5 showed excellent binding activities to SARS-CoV-2 and SARS-CoV NPs in the flow cytometry analysis (Fig. 2b). To test the application of P301-F7 in the cellular localization of NP in SARS-CoV-2 infected cells, we performed the immunofluorescence assay (IFA) to evaluate the inhibition effect of Remdesivir in live virus infected Vero E6 cells (Fig. 2c). The results showed sufficient fluorescence intensity and a good dose response pattern, indicating that P301-F7 could be considered as a kind of NP-specific detection antibody in various immunofluorescence assays. SARS-CoV-2 spike-specific nAbs are good candidates for preventing and treating COVID-19. Antibody neutralization capacity against live virus is the most important evaluation index of nAbs. Currently, the ability of nAbs to inhibit the infection of SARS-CoV-2 live virus has been evaluated by the FRNT [5, 19], in which some commercial polyclonal antibodies or mAbs against NP are used to detect the target cells infected by live virus. Therefore, we labeled P301-F7 with HRP and then tested its ability to recognize the infected Vero E6 cells. The results showed that the nonspecific background of P301-F7-HRP was very low, yet the positive signal was particularly clear and similar to our previous assay [5] and possessed a good virus-dosage effect (Fig. 2d). Finally, as shown in Fig. 2e and Additional file 1: Figure S4, we successfully applied the P301-F7-HRP to evaluate the neutralization of a nAb, P2C-1F11, whose 50% inhibition concentration (IC_50_) was calculated as 0.03 μg/mL and similar to that previously reported [5].

**Fig. 3:**
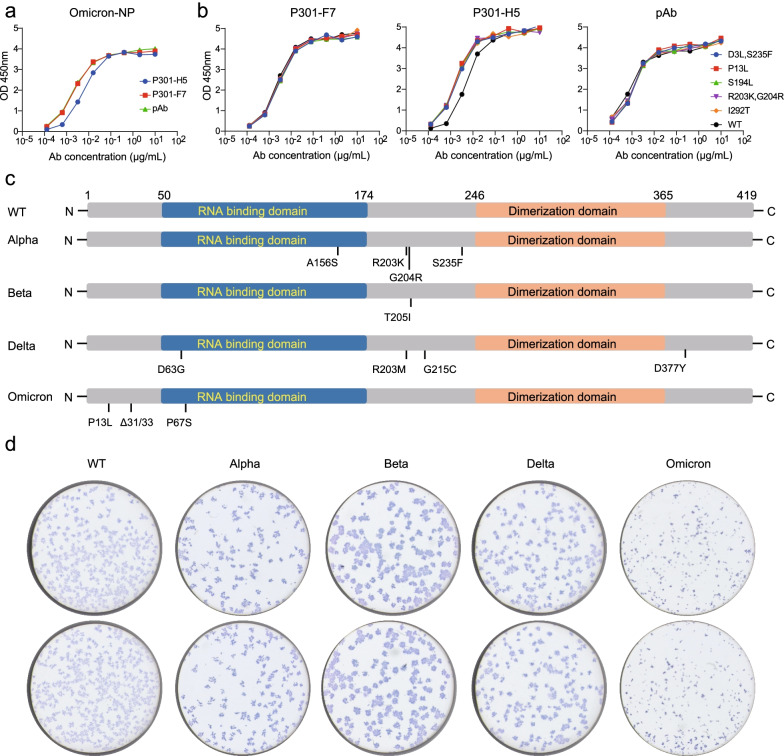
The abilities of P301-F7 and P301-H5 in recognizing the mutated NPs and live viruses of SARS-CoV-2 variants. ELISA analysis of the binding activities of P301-F7 and P301-H5 to the SARS-CoV-2 Omicron NP (**a**) and the mutated NPs (**b**) carrying D3L/S235F, P13L, S194L, R203K/G204R, and I292T substitutions, respectively. The pAb is used here as a positive control. **c** Key mutations appear in the NP of isolated SARS-CoV-2 live viruses used in this study. **d** The immune spot assay analysis of the recognition abilities of P301-F7 to the WT SARS-CoV-2 and variants (Alpha, Beta, Delta, and Omicron). The Vero E6 cells were infected with SARS-CoV-2 live virus, respectively, and then stained by the HRP-labeled P301-F7. The experiment in **a**, **b**, **d** was performed twice and one representative result was shown

In addition, we tested the binding activities of P301-F7 and P301-H5 to the SARS-CoV-2 Omicron and other mutated NPs carrying D3L/S235F, P13L, S194L, R203K/G204R, and I292T mutations, respectively (Fig. 3a, b). Both mAbs still bound well to these mutated proteins with similar activities to the WT NP, indicating that P301-F7 and P301-H5 might be broadly binding antibodies. Of note, we further tested the ability of P301-F7-HRP in recognizing variant live viruses of SARS-CoV-2 including Alpha, Beta, Delta, and Omicron, and the comparison between their mutated amino acids with the WT was listed in Fig. 3c and Additional file 1: Figure S5. Vero E6 cells infected by WT live viruses and variants were clearly displayed by the immune spot assay mediated by P301-F7-HRP (Fig. 3d), whose broadly binding activity to SARS-CoV-2 variants would contribute its potentially wider application in the antigen detection. We also investigated whether P301-F7 and P301-H5 recognize NPs of other existing human coronaviruses including HKU1, OC43, NL63, and 229E. The results showed that these two mAbs did not react with the NPs of these viruses (Additional file 1: Figure S6), which would reduce the false positive rate in the detection of SARS-CoV-2 variants.

## Discussion

Compared with spike protein, NP is normally conserved and highly immunogenic in many coronavirus members. It has been reported that antibodies to the NP of SARS-CoV-2 are more sensitive than the spike protein specific antibodies for early infection detection [20]. In addition, immunoglobulin M (IgM) and IgG against SARS-CoV-2 NP have been successfully detected in serological testing and epidemiological monitoring [21–23]. In the COVID-19 patients, viral antigen detection was also a convenient method to directly demonstrate the infection of SARS-CoV-2 as compared with nucleic acid amplification tests (NATs), which could provide test results in a much shorter time span under 30 minutes [15]. The key agents of exploring detection assay are antibodies specifically binding to NP antigen. However, the uneven composition of polyclonal antibodies may affect the accuracy of experimental results. At present, a number of NP-specific mAbs with different epitopes, cross-reactivity and affinity have been isolated from mice, rabbits, alpacas and convalescence patients, indicating that they can be applied to different detection scenarios of SARS-CoV-2 [14–16, 21, 24]. Terry et al. developed a series of mouse mAbs against the SARS-CoV-2 NP. Similar to our results, most of mAbs could also recognize the SARS-CoV NP, except mBG86, which was a SARS-CoV-2 specific mAb. The epitope of mBG17 was located in the region of aa381-419 at the C-terminal of NP close to the epitope of P301-H5 (aa393-407). This pair of noncompeting mAbs, mBG17 and mBG86, recognizing distinct epitopes could be used in the sandwich ELISA. The pity of it was that the binding kinetics and affinities of these mouse mAbs were not analyzed. Gransagne et al. reported seven single variable domain on heavy chain (VHH) antibodies isolated from alpaca that mainly targeted conformational epitopes in either the C-terminal or the N-terminal domains. Just like P301-F7 and P301-H5, these VHHs did not react with NPs of another four common human coronaviruses. Their binding affinities to SARS-CoV-2 NP ranged from 0.206 to 46.5 nM. However, it’s better to make a head-to-head comparison with other mAbs, because the affinity analysis might exist differences in separate laboratories. And for those commercial mAbs, they are not only expensive, but also limited in the development of product reagents due to the unknown characteristic information and lack of independent intellectual property rights. In this study, we provided two human mAbs crossly recognizing NPs of SARS-CoV-2 variants including Omicron and even SARS-CoV. More notably, P301-F7 and P301-H5 bound to two distinct epitopes and could be used in multiple biochemical assays for the detection of NP antigen. These two novel noncompeting mAbs could be used alone or in combination, which would promote the development of diagnosis of SARS-CoV-2.

In the past two years, SARS-CoV-2 was still spreading worldwide, especially with the continuous appearance of variants. For example, the Delta variant was first identified in October 2020 in India and has since become a long-term dominant in the global COVID-19 pandemic [8, 25]. Nonetheless, a novel variant, Omicron, carried an unprecedented number of mutations and had spread more rapidly to over 60 countries due to the enhanced transmissibility [26]. The mutations appeared in the region of NP may affect the availability of the existing antigen detection agents for testing these SARS-CoV-2 variants. So far as we know, P301-F7 is the first NP-specific mAb which has been proved to be able to recognize Vero E6 cells infected with the live viruses of variants including Alpha, Beta, Delta, and Omicron. Due to the limited resource of the biosafety level 3 lab, we first evaluated the ability of P301-F7 to recognize SARS-CoV-2 WT live viruses and variants. ELISA results showed that P301-H5 could also effectively bind to the WT and mutated NPs including Omicron, and that the P99 peptide (393-TLLPAADLDDFSKQL-407) was the key epitope of P301-H5. In this study, We tested the application of P301-F7 and P301-H5 in biological experiments such as ELISA, WB, and flow cytometry in parallel. These data suggest that P301-H5 may also be used in the detection of SARS-CoV-2 live virus. Overall, P301-F7 and P301-H5 reported here can serve as good candidates for developing the precise antigen detection kits for reliable diagnosis of SARS-CoV-2 variants and even SARS-CoV-like viruses.

## Supplementary Information

**Additional file 1.** **Figure S1.** The isolation of a pair of noncompeting mAbs binding to SARS-CoV-2 and SARS-CoV NPs. (a) The binding activity of plasma from a convalescent COVID-19 patient to SARS-CoV-2 NP measured by ELISA. Patient: P301. HD: Healthy donor. (b) The gating strategy for isolation of SARS-CoV-2 WT NP-specific B cells by FACS. (c) The binding activities of five mAbs to NPs of SARS-CoV-2, SARS-CoV, and MERS-CoV, respectively, measured by ELISA. The pAb is used here as a positive control. The IgG1 is a negative control. (d) Competition ELISA of P301-F7 with itself and P301-H5. The IgG1 is a negative control. **Figure S2.** The identification of epitopes recognized by P301-F7 and P301-H5. (a) ELISA binding of P301-F7 and P301-H5 to native and denatured SARS-CoV-2 NP which was treated with the denaturing buffer containing 0.5% SDS and 40 mM DTT1 (New England Biolabs) at 100 °C for 10 mins. The pAb is used here as a positive control. VRC01 recognizing conformational epitopes of HIV-1 gp140 was served as negative control to prove that the denaturing process was efficient. (b) Construction strategies of expression vectors of the full-length and two C-terminal truncated SARS-CoV-2 NPs. NP: M1-A419. NP-T1: M1-E280. NP-T2: M1-M210. (c) The full-length and two C-terminal truncated SARS-CoV-2 NPs were expressed in 293 T cells by the transient transfection, respectively, and then detected by P301-F7 and P301-H5 using the flow cytometry analysis. The pAb is used here as a positive control. (d) The recognition epitope of P301-F7 and P301-H5 were identified by ELISA screening of peptide pools of SARS-CoV-2 NP. **Figure S3.** The sandwich ELISA detection of SARS-CoV and SARS-CoV-2 NPs using P301-F7 and HRP conjugated P301-H5. P301-F7 is used as a capture antibody. P301-H5-HRP is used as a detection antibody. **Figure S4.** The neutralizing activity of P2C-1F11 against SARS-CoV-2 WT live virus was measured by the FRNT. The IgG1 is a negative control. P301-F7-HRP is used as a detection antibody. **Figure S5.** Key mutations appear in the NP of SARS-CoV-2 variant viruses. Amino acid mutations of NPs in SARS-CoV-2 variants according to GISAID EpiCoV database (https://www.gisaid.org/hcov19-mutationdashboard/) and outbreak.info. **Figure S6.** ELISA binding of P301-F7 and P301-H5 to NPs of endemic coronaviruses including HKU1, OC43, NL63 and 229E. The pAb is used here as a positive control.

## Data Availability

We are happy to share reagents and information in this study upon request.

## Abbreviations

SARS-CoV-2: Severe acute respiratory syndrome coronavirus 2
NP: Nucleocapsid protein
ACE2: Angiotensin-converting enzyme 2
mAbs: Monoclonal antibodies
nAb: Neutralizing antibody
WT: Wild type

## References

[1] Zhu N (2020). A novel coronavirus from patients with pneumonia in China, 2019. N Engl J Med.

[2] Drosten C (2003). Identification of a novel coronavirus in patients with severe acute respiratory syndrome. N Engl J Med.

[3] Zaki AM, van Boheemen S, Bestebroer TM, Osterhaus AD, Fouchier RA (2012). Isolation of a novel coronavirus from a man with pneumonia in Saudi Arabia. N Engl J Med.

[4] Lu R (2020). Genomic characterisation and epidemiology of 2019 novel coronavirus: implications for virus origins and receptor binding. Lancet (London, England).

[5] Ju B (2020). Human neutralizing antibodies elicited by SARS-CoV-2 infection. Nature.

[6] Wang R (2021). Analysis of SARS-CoV-2 variant mutations reveals neutralization escape mechanisms and the ability to use ACE2 receptors from additional species. Immunity.

[7] Wang P (2021). Antibody resistance of SARS-CoV-2 variants B.1.351 and B.1.1.7. Nature.

[8] Liu C (2021). Reduced neutralization of SARS-CoV-2 B.1.617 by vaccine and convalescent serum. Cell.

[9] Cheng L (2021). Cross-neutralization of SARS-CoV-2 Kappa and Delta variants by inactivated vaccine-elicited serum and monoclonal antibodies. Cell Discov.

[10] Wu H (2021). Nucleocapsid mutations R203K/G204R increase the infectivity, fitness, and virulence of SARS-CoV-2. Cell Host Microbe.

[11] Syed AM (2021). Rapid assessment of SARS-CoV-2 evolved variants using virus-like particles. Science.

[12] Liu SJ (2006). Immunological characterizations of the nucleocapsid protein based SARS vaccine candidates. Vaccine.

[13] Dutta NK, Mazumdar K, Gordy JT (2020). The nucleocapsid protein of SARS-CoV-2: a target for vaccine development. J Virol.

[14] Terry JS (2021). Development of a SARS-CoV-2 nucleocapsid specific monoclonal antibody. Virology.

[15] Yamaoka Y (2021). Highly specific monoclonal antibodies and epitope identification against SARS-CoV-2 nucleocapsid protein for antigen detection tests. Cell Rep Med.

[16] Tian X (2021). Epitope mapping of severe acute respiratory syndrome-related coronavirus nucleocapsid protein with a rabbit monoclonal antibody. Virus Res.

[17] Kang S (2021). A SARS-CoV-2 antibody curbs viral nucleocapsid protein-induced complement hyperactivation. Nat Commun.

[18] Ju B (2018). Identification of a novel broadly HIV-1-neutralizing antibody from a CRF01_AE-infected Chinese donor. Emerg Microbes Infect.

[19] Zhang Q (2021). Potent and protective IGHV3-53/3-66 public antibodies and their shared escape mutant on the spike of SARS-CoV-2. Nat Commun.

[20] Burbelo PD (2020). Sensitivity in detection of antibodies to nucleocapsid and spike proteins of severe acute respiratory syndrome coronavirus 2 in patients with coronavirus disease 2019. J Infect Dis.

[21] Zhang L (2020). Development of patient-derived human monoclonal antibodies against nucleocapsid protein of severe acute respiratory syndrome coronavirus 2 for coronavirus disease 2019 diagnosis. Front Immunol.

[22] Xie C (2022). Preparation of highly specific monoclonal antibodies against SARS-CoV-2 nucleocapsid protein and the preliminary development of antigen detection test strips. J Med Virol.

[23] Zeng W (2020). Biochemical characterization of SARS-CoV-2 nucleocapsid protein. Biochem Biophys Res Commun.

[24] Gransagne M (2022). Development of a highly specific and sensitive VHH-based sandwich immunoassay for the detection of the SARS-CoV-2 nucleoprotein. J Biol Chem.

[25] Planas D (2021). Reduced sensitivity of SARS-CoV-2 variant Delta to antibody neutralization. Nature.

[26] Liu L (2021). Striking antibody evasion manifested by the Omicron variant of SARS-CoV-2. Nature.

