# Supplementary material for: Identification and application of a pair of noncompeting monoclonal antibodies broadly binding to the nucleocapsid proteins of SARS-CoV-2 variants including Omicron
Source: Virol J. 2022 May 28;19:96. doi: 10.1186/s12985-022-01827-w (PMC9142731; doi:10.1186/s12985-022-01827-w)
Supplement: Supplementary file 1 — Additional file 1. Figure S1. The isolation of a pair of noncompeting mAbs binding to SARS-CoV-2 and SARS-CoV NPs. (a) The binding activity of plasma from a convalescent COVID-19 patient to SARS-CoV-2 NP measured by ELISA. Patient: P301. HD: Healthy donor. (b) The gating strategy for isolation of SARS-CoV-2 WT NP-specific B cells by FACS. (c) The binding activities of five mAbs to NPs of SARS-CoV-2, SARS-CoV, and MERS-CoV, respectively, measured by ELISA. The pAb is used here as a positive control. The IgG1 is a negative control. (d) Competition ELISA of P301-F7 with itself and P301-H5. The IgG1 is a negative control. Figure S2. The identification of epitopes recognized by P301-F7 and P301-H5. (a) ELISA binding of P301-F7 and P301-H5 to native and denatured SARS-CoV-2 NP which was treated with the denaturing buffer containing 0.5% SDS and 40 mM DTT1 (New England Biolabs) at 100 °C for 10 mins. The pAb is used here as a positive control. VRC01 recognizing conformational epitopes of HIV-1 gp140 was served as negative control to prove that the denaturing process was efficient. (b) Construction strategies of expression vectors of the full-length and two C-terminal truncated SARS-CoV-2 NPs. NP: M1-A419. NP-T1: M1-E280. NP-T2: M1-M210. (c) The full-length and two C-terminal truncated SARS-CoV-2 NPs were expressed in 293 T cells by the transient transfection, respectively, and then detected by P301-F7 and P301-H5 using the flow cytometry analysis. The pAb is used here as a positive control. (d) The recognition epitope of P301-F7 and P301-H5 were identified by ELISA screening of peptide pools of SARS-CoV-2 NP. Figure S3. The sandwich ELISA detection of SARS-CoV and SARS-CoV-2 NPs using P301-F7 and HRP conjugated P301-H5. P301-F7 is used as a capture antibody. P301-H5-HRP is used as a detection antibody. Figure S4. The neutralizing activity of P2C-1F11 against SARS-CoV-2 WT live virus was measured by the FRNT. The IgG1 is a negative control. P301-F7-HRP is used as a detectio [file 12985_2022_1827_MOESM1_ESM.docx]

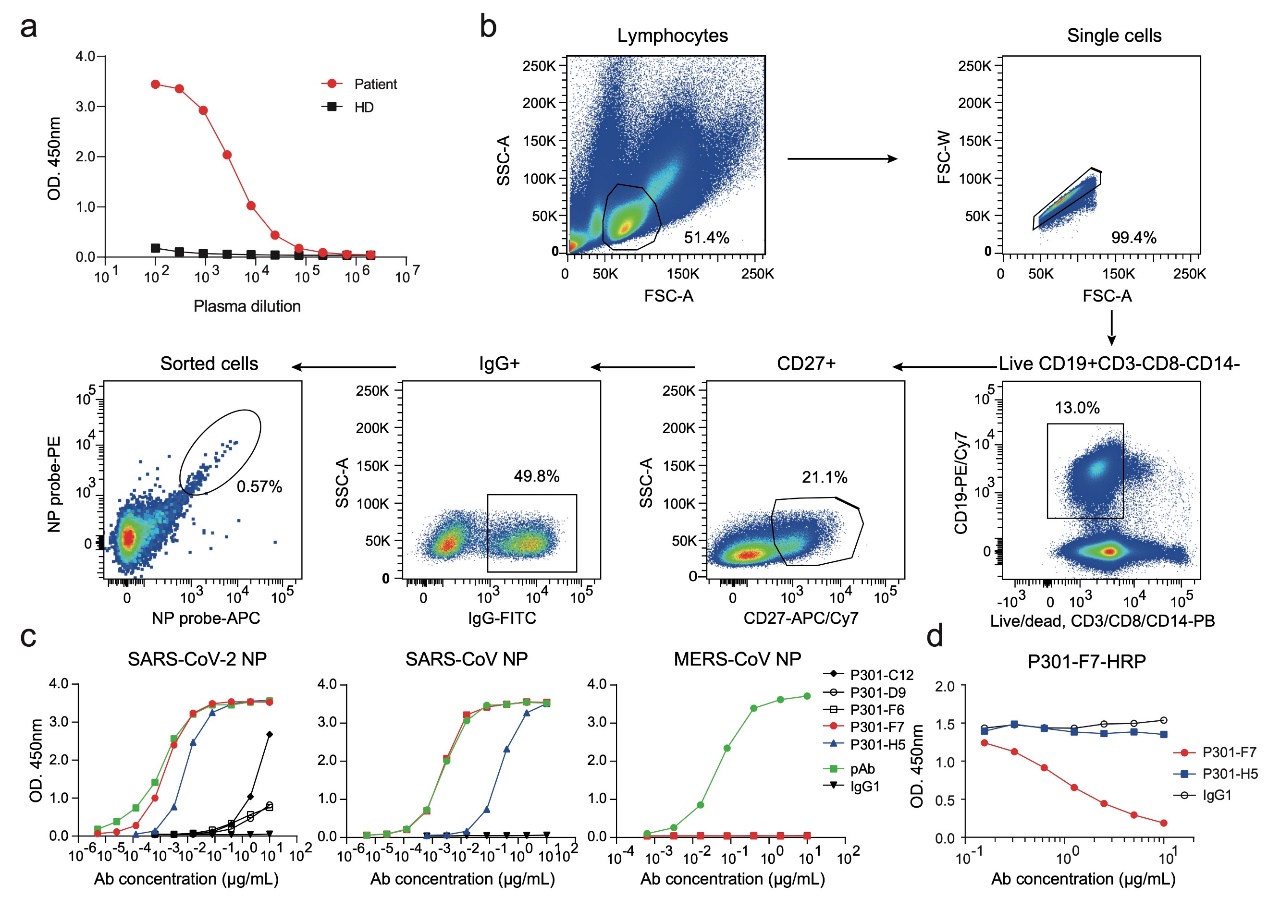


**Figure S1. The isolation of a pair of noncompeting mAbs binding to SARS-CoV-2 and SARS-CoV NPs.**

**(a)** The binding activity of plasma from a convalescent COVID-19 patient to SARS-CoV-2 NP measured by ELISA. Patient: P301. HD: Healthy donor. **(b)** The gating strategy for isolation of SARS-CoV-2 WT NP-specific B cells by FACS. **(c)** The binding activities of five mAbs to NPs of SARS-CoV-2, SARS-CoV, and MERS-CoV, respectively, measured by ELISA. The pAb is used here as a positive control. The IgG1 is a negative control. **(d)** Competition ELISA of P301-F7 with itself and P301-H5. The IgG1 is a negative control.


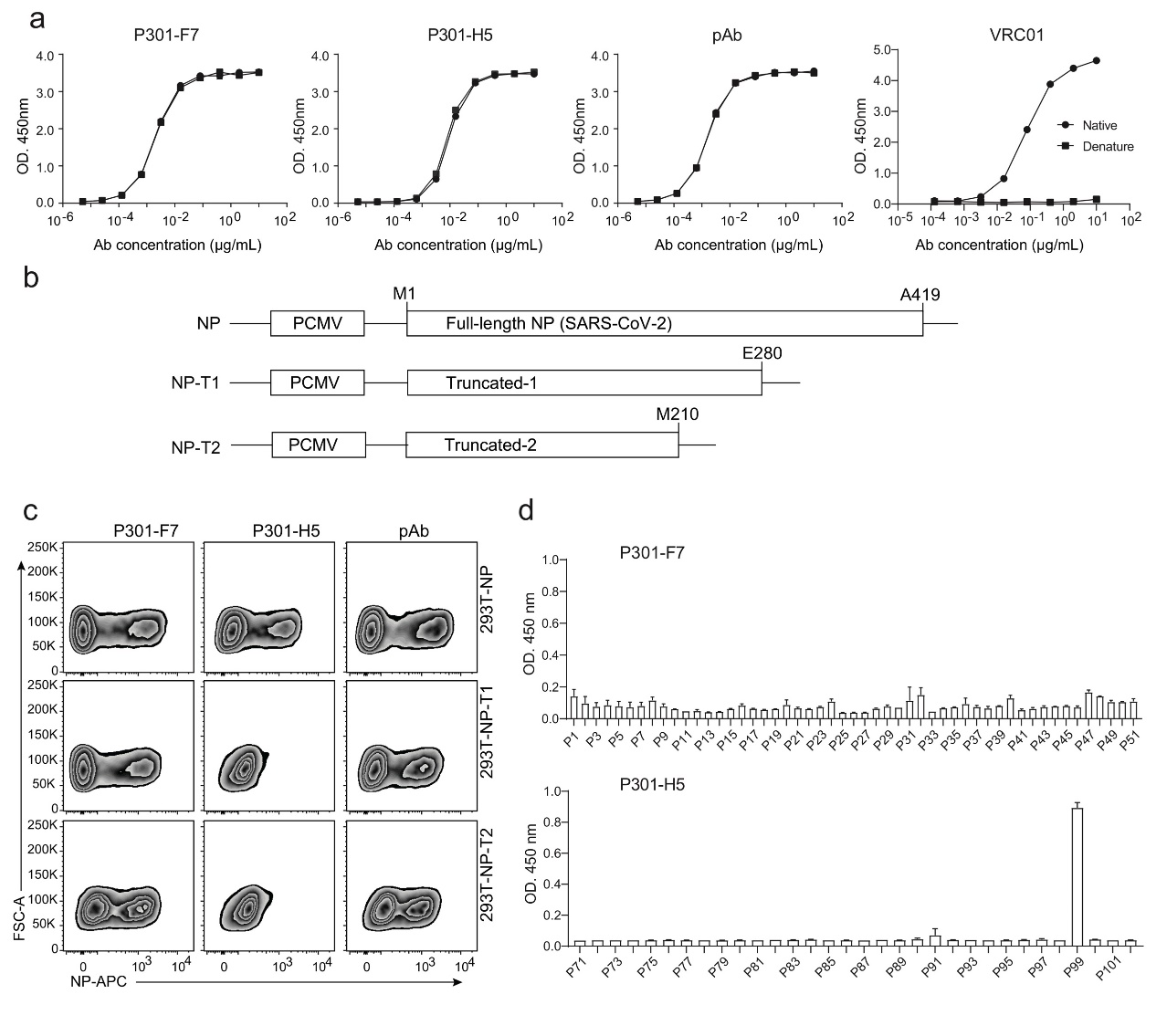


**Figure S2. The identification of epitopes recognized by P301-F7 and P301-H5.**

**(a)** ELISA binding of P301-F7 and P301-H5 to native and denatured SARS-CoV-2 NP which was treated with the denaturing buffer containing 0.5% SDS and 40 mM DTT^1^ (New England Biolabs) at 100 °C for 10 mins. The pAb is used here as a positive control. VRC01 recognizing conformational epitopes of HIV-1 gp140 was served as negative control to prove that the denaturing process was efficient. **(b)** Construction strategies of expression vectors of the full-length and two C-terminal truncated SARS-CoV-2 NPs. NP: M1-A419. NP-T1: M1-E280. NP-T2: M1-M210. **(c)** The full-length and two C-terminal truncated SARS-CoV-2 NPs were expressed in 293 T cells by the transient transfection, respectively, and then detected by P301-F7 and P301-H5 using the flow cytometry analysis. The pAb is used here as a positive control. **(d)** The recognition epitope of P301-F7 and P301-H5 were identified by ELISA screening of peptide pools of SARS-CoV-2 NP.


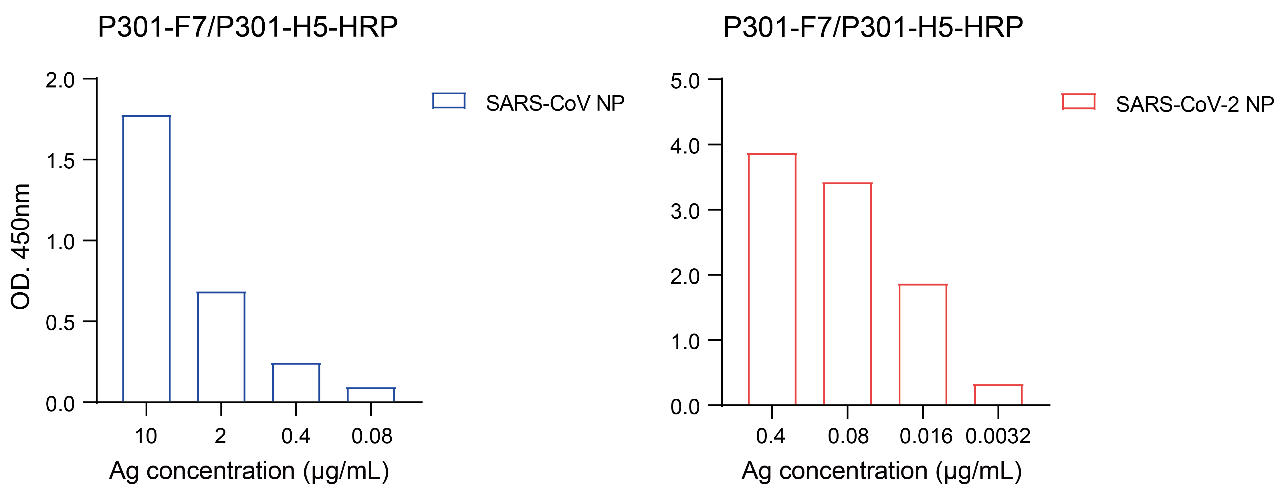


**Figure S3. The sandwich ELISA detection of SARS-CoV and SARS-CoV-2 NPs using P301-F7 and HRP conjugated P301-H5.**

P301-F7 is used as a capture antibody. P301-H5-HRP is used as a detection antibody.


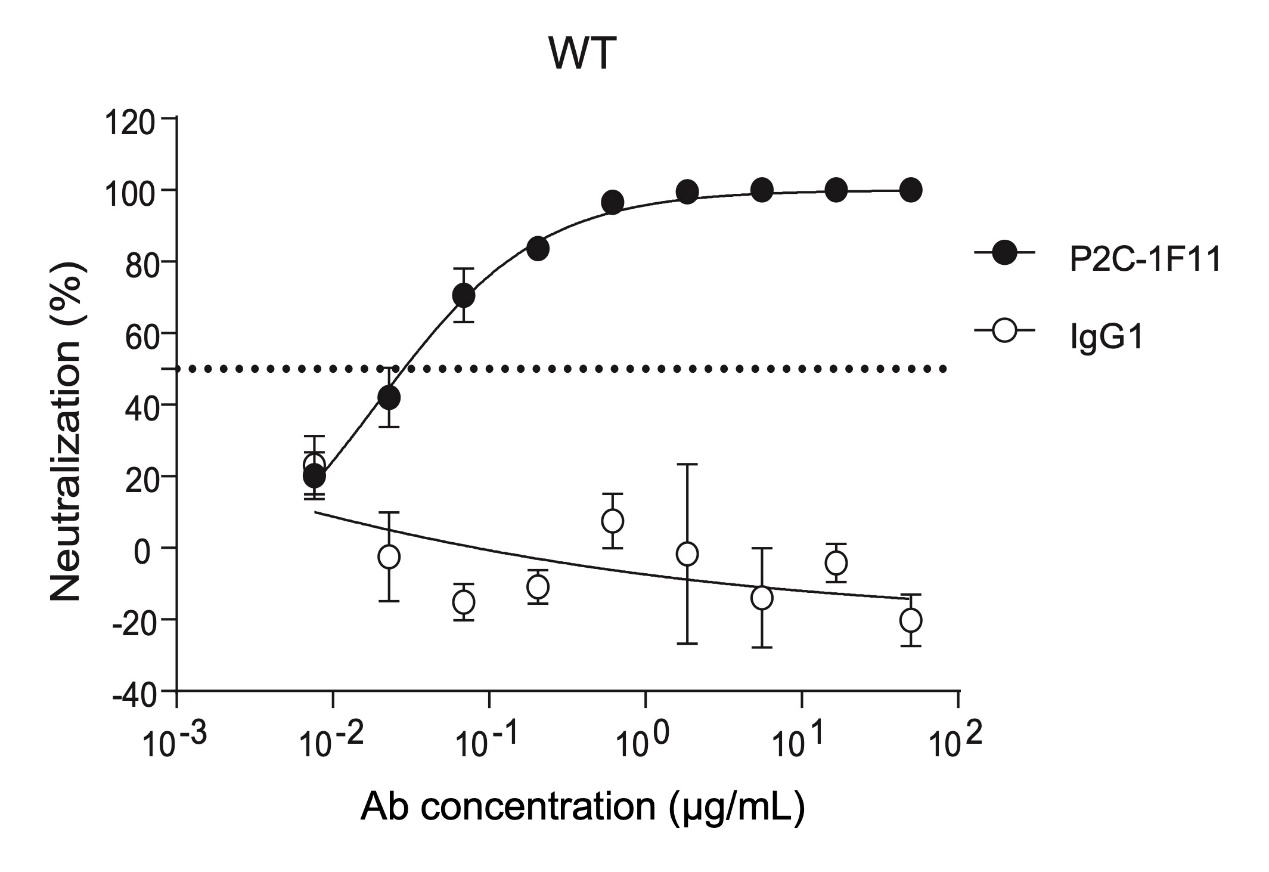


**Figure S4. The neutralizing activity of P2C-1F11 against SARS-CoV-2 WT live virus was measured by the FRNT.**

The IgG1 is a negative control. P301-F7-HRP is used as a detection antibody.


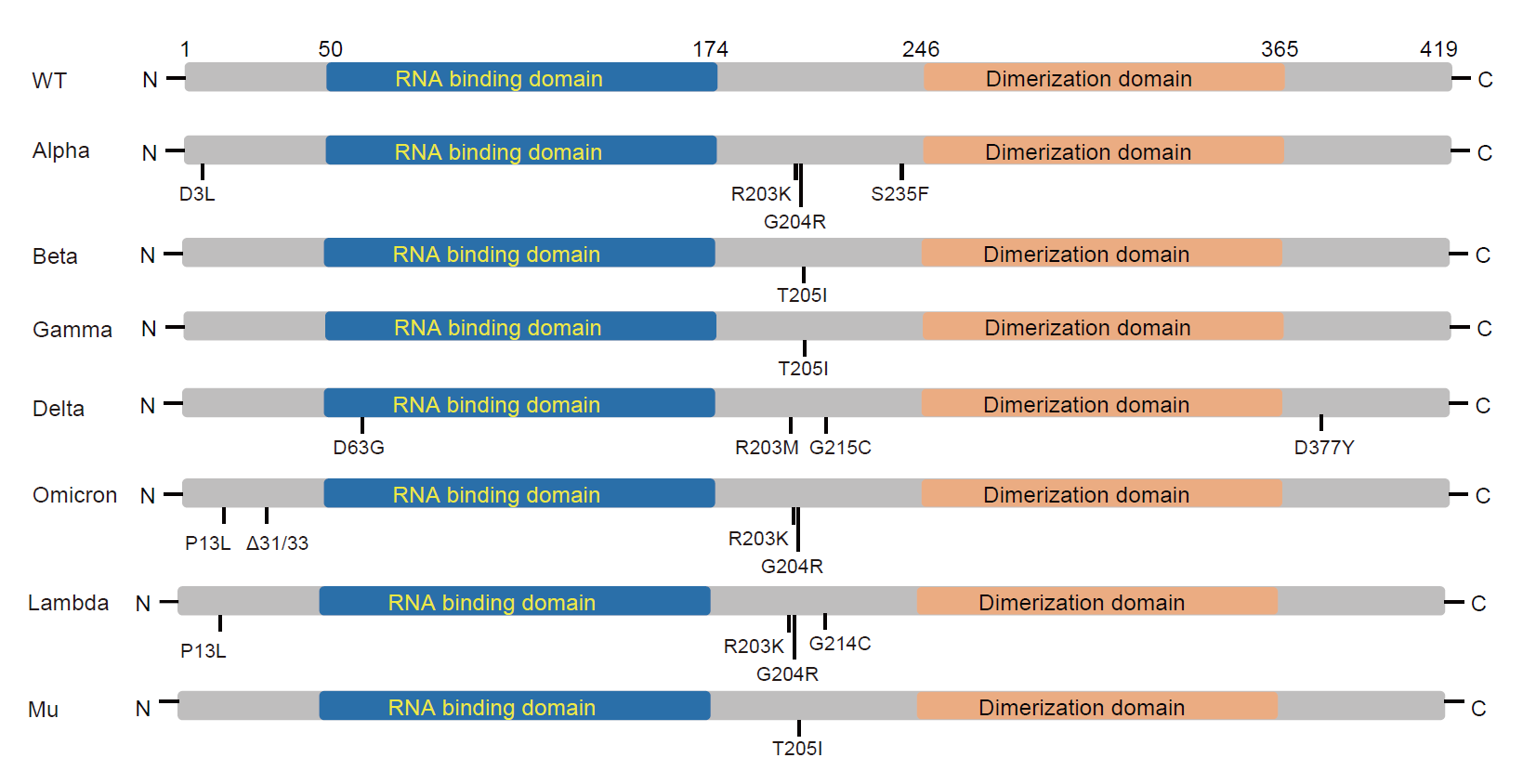
 **Figure S5. Key mutations appear in the NP of SARS-CoV-2 variant viruses.**

Amino acid mutations of NPs in SARS-CoV-2 variants according to GISAID EpiCoV database (https://www.gisaid.org/hcov19-mutation-dashboard/) and outbreak.info.


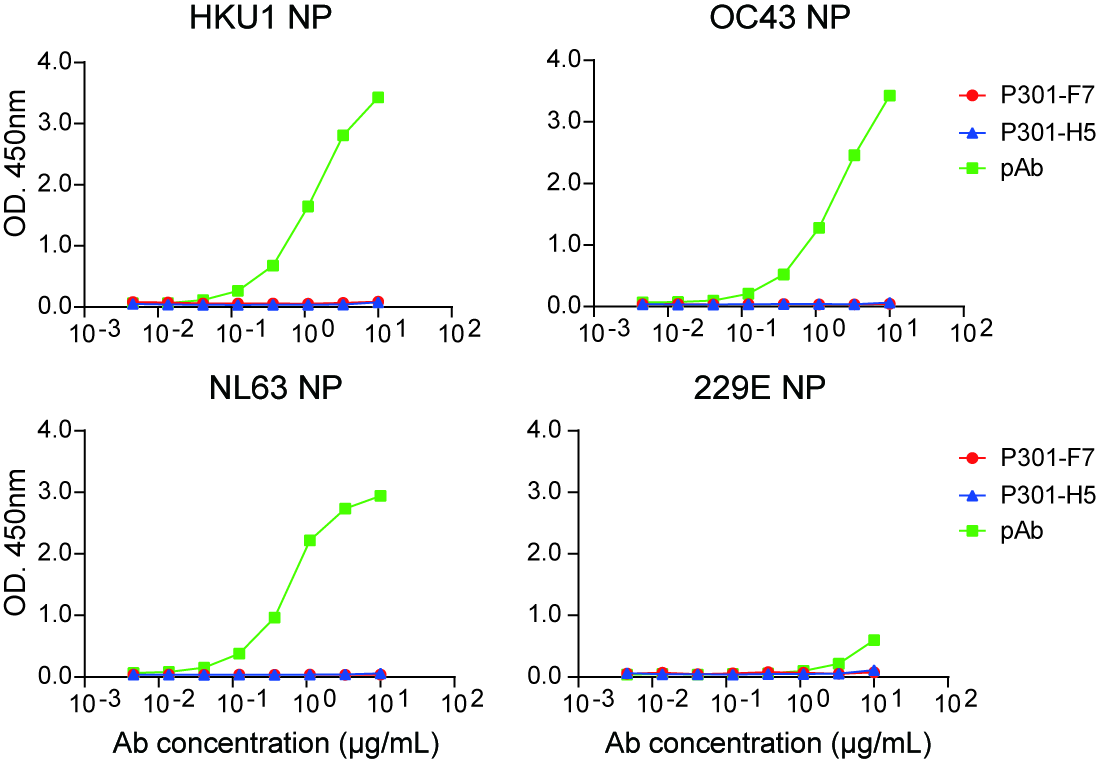


**Figure S6. ELISA binding of P301-F7 and P301-H5 to NPs of endemic coronaviruses including HKU1, OC43, NL63 and 229E.**

The pAb is used here as a positive control.

**References**

1 Ju, B. *et al.* Identification of a novel broadly HIV-1-neutralizing antibody from a CRF01_AE-infected Chinese donor. *Emerg Microbes Infect* **7**, 174, doi:10.1038/s41426-018-0175-1 (2018).
